# Open Repair of a Giant Superficial Femoral-Popliteal Artery Aneurysm Using Autologous Saphenous Vein Bypass: A Case Report

**DOI:** 10.7759/cureus.115222

**Published:** 2026-08-26

**Authors:** Mohammed Y Aldossary, Talal A Alzaharani, Abdulrahman A AlRubayyi, Sarah F Alkhan

**Affiliations:** 1 Department of General Surgery and Vascular Surgery, Security Forces Hospital, Dammam, SAU; 2 Department of General Surgery, Security Forces Hospital, Dammam, SAU; 3 Department of Family Medicine, Security Forces Hospital, Dammam, SAU

**Keywords:** autologous saphenous vein graft, open surgical repair, peripheral arterial aneurysms, popliteal artery aneurysm, superficial femoral artery aneurysm

## Abstract

Peripheral arterial aneurysms are rare but clinically significant because of their risk of thromboembolism and limb-threatening ischemia. Popliteal artery aneurysms are the most common peripheral aneurysms, whereas superficial femoral artery aneurysms are uncommon and often diagnosed late. We report the case of a 60-year-old man who presented with a two-week history of intermittent left thigh pain associated with coldness and numbness. Examination revealed absent distal pulses. CT angiography (CTA) demonstrated a giant superficial femoral-popliteal fusiform aneurysm measuring 7.1 × 5.8 cm and 10 cm in length, with mural thrombosis and preserved distal runoff. The patient underwent aneurysm exclusion and bypass from the mid superficial femoral artery to the distal popliteal artery using a non-reversed in situ autologous great saphenous vein (GSV) graft. Recovery was uneventful, and six-month follow-up confirmed a patent graft with persistent thrombosis of the excluded aneurysm. Early recognition and CTA are essential for diagnosis and operative planning. Open repair with an autologous GSV remains a durable treatment option with excellent long-term outcomes in appropriately selected patients. This case highlights the importance of early diagnosis and timely surgical intervention in achieving successful limb salvage and durable revascularization.

## Introduction

Peripheral arterial aneurysms are uncommon compared with aortic aneurysms but remain clinically significant because of their association with thromboembolic events and limb-threatening ischemia. An arterial aneurysm is defined as a localized dilation of a vessel to at least 1.5 times its normal diameter [1]. Among peripheral aneurysms, popliteal artery aneurysms (PAAs) are the most common, accounting for approximately 70-85% of cases, with an estimated prevalence of about 1% in older men [2,3]. In contrast, true superficial femoral artery aneurysms (SFAAs) are rare and represent a small proportion of peripheral arterial aneurysms. Nevertheless, contemporary case series have demonstrated that these lesions are encountered in vascular practice, although much less frequently than PAAs [4-8]. The estimated incidence of true SFAAs is approximately eight per 100,000 individuals, with fewer than 200 cases reported in the literature, underscoring the uncommon nature of this vascular pathology [8].

Both conditions are strongly associated with atherosclerosis and share common risk factors, such as advanced age, male sex, smoking, and hypertension [1,5]. Although combined superficial femoral-popliteal aneurysms are recognized in vascular practice, the present case is educational because of the large aneurysm size, chronic ischemic presentation despite preserved distal runoff, and successful open reconstruction using an autologous great saphenous vein (GSV) bypass.

This case report aims to present the clinical features and management considerations of superficial femoral and PAAs, emphasizing early recognition and timely intervention to reduce morbidity. It has been reported in line with the SCARE checklist [9].

## Case presentation

A 60-year-old man with a history of asthma presented to the emergency department with a two-week history of left thigh pain. The pain was intermittent, sudden in onset, and associated with coldness and numbness in the same limb. It was aggravated by walking and prolonged standing and relieved by rest. The patient denied any history of abdominal pain or pain in the contralateral limb. His past medical, surgical, and family histories were otherwise unremarkable.

On physical examination, the patient was in mild discomfort but was hemodynamically stable and afebrile. His blood pressure was 131/78 mmHg, heart rate was 83 beats/min, respiratory rate was 21 breaths/min, temperature was 37.1 °C, and oxygen saturation was 99% on room air (Table 1). Distal pulses in the left foot were not palpable, and no masses were detected.

**Table 1:** Admission vital signs and laboratory investigations.

Parameter	Patient value	Unit	Reference range
Blood pressure	131/78	mmHg	90-120/60-80 mmHg
Heart rate	83	beats/min	60-100 beats/min
Respiratory rate	21	breaths/min	12-20 breaths/min
Temperature	37.1	°C	36.0-37.5 °C
Oxygen saturation	99	%	95-100%
Hemoglobin	13.2	g/dL	13.0-17.0 g/dL
Leukocyte count	5.71	× 10⁹/L	4.0-10.0 × 10⁹/L
Platelet count	203	× 10⁹/L	150-400 × 10⁹/L
D-dimer	1.79	µg/mL	<0.50 µg/mL FEU
C-reactive protein	3.5	mg/L	<5 mg/L
Erythrocyte sedimentation rate	16	mm/h	0-15 mm/h
Creatinine	0.74	mg/dL	0.7-1.3 mg/dL
Urea	3.2	mmol/L	2.5-7.1 mmol/L
Aspartate aminotransferase	27	U/L	10-40 U/L
Alanine aminotransferase	41	U/L	7-56 U/L
Total bilirubin	0.5	mg/dL	0.2-1.2 mg/dL
Prothrombin time/international normalized ratio	0.9	-	0.8-1.2
Activated partial thromboplastin time	29	seconds	25-35 seconds

Differential diagnoses considered at presentation included peripheral arterial occlusive disease, acute arterial embolism, deep vein thrombosis, and musculoskeletal causes of thigh pain. However, the absence of distal pulses, together with symptoms of intermittent ischemia manifested by pain, coldness, and numbness, raised strong suspicion of an underlying arterial pathology. The lack of limb swelling or clinical features suggestive of venous disease, along with the absence of trauma or musculoskeletal findings, made these alternative diagnoses less likely.

**Figure 1:**
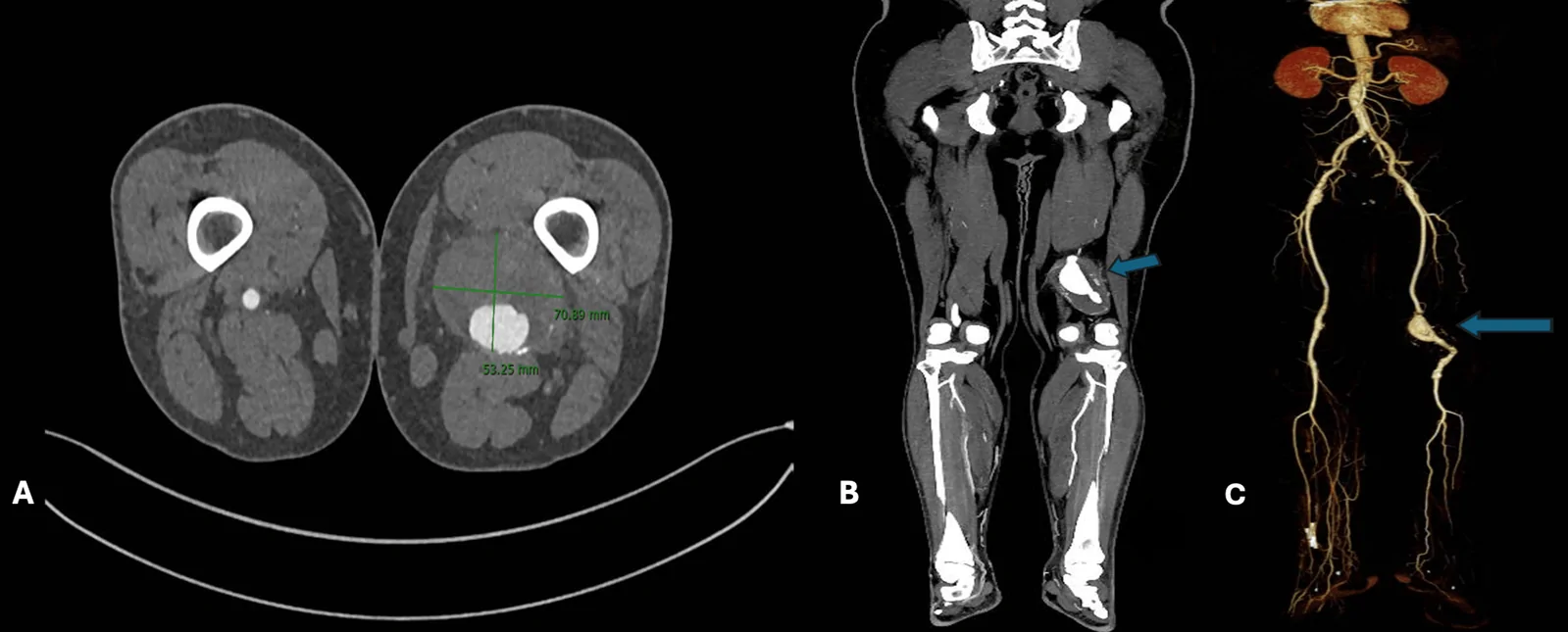
CTA of the left lower limb. (A) Axial image demonstrating a giant superficial femoral-popliteal fusiform aneurysm with mural calcification and circumferential thrombus, measuring 7.1 × 5.8 cm (arrowhead). (B) Coronal reconstruction showing the longitudinal extent of the aneurysm (approximately 10 cm) (arrowhead). (C) Three-dimensional CTA reconstruction illustrating the aneurysm and its anatomical relationship to the superficial femoral and popliteal arteries (arrowhead). CTA, CT angiography

Consequently, CT angiography (CTA) was performed to establish the diagnosis, define the extent of the lesion, assess distal runoff, and guide surgical planning. It revealed a left-sided superficial femoral-popliteal fusiform aneurysm with wall calcifications, measuring 7.1 × 5.8 cm in axial dimensions and 10 cm in length, with circumferential mural thrombosis (Figure 1A-1C). The lumen remained patent, with preserved distal arterial runoff. No abdominal or contralateral limb aneurysms were identified.

Laboratory investigations showed a hemoglobin level of 13.2 g/dL, leukocyte count of 5.71 × 10⁹/L, platelet count of 203 × 10⁹/L, and D-dimer level of 1.79 µg/mL. C-reactive protein, liver and renal function tests, erythrocyte sedimentation rate, and coagulation profile were within normal limits (Table 1).

The patient was scheduled for open surgical repair under spinal anesthesia. Through combined upper and lower medial approaches, the aneurysm was exposed. The patient was positioned supine with the knee flexed over a sandbag. The upper medial incision extended from approximately four fingerbreadths above the adductor canal downward and posteriorly toward the medial femoral condyle, taking care to preserve the GSV. The deep fascia was incised, and the sartorius muscle was retracted posteriorly to expose the adductor canal. A lower medial approach was then performed along the posterior border of the tibia, with retraction of the medial head of the gastrocnemius muscle to expose the popliteal vessels. The popliteal artery and vein were carefully dissected.

A bypass was performed from the mid superficial femoral artery to the distal popliteal artery (below the knee) using a non-reversed in situ autologous GSV graft with a valvulotome. The anastomoses were performed in an end-to-side fashion. The aneurysm was excluded by proximal and distal ligation using polypropylene sutures. Following revascularization, strong triphasic signals were obtained in the posterior tibial and dorsalis pedis arteries, and the aneurysm sac lost its pulsatility.

A non-reversed in situ GSV graft was selected because the ipsilateral vein was suitable and could be preserved in its anatomical bed, allowing better diameter matching between the proximal superficial femoral artery and distal popliteal artery. This approach also avoided complete vein harvest and reversal while providing an autologous conduit with favorable handling characteristics. The valves were disrupted using a valvulotome to ensure unobstructed arterial flow.

The postoperative course was uneventful. The patient was able to ambulate on postoperative day 3 and was discharged on postoperative day 12 without the need for assistive devices. The patient was started on aspirin and low-dose rivaroxaban according to the institutional vascular surgery protocol. At the six-month follow-up, the patient remained asymptomatic, with no recurrent ischemic symptoms. Follow-up was scheduled with clinical assessment of symptoms, distal pulse examination, and graft surveillance using duplex ultrasonography. CTA was reserved for cases of suspected graft stenosis or occlusion, recurrent symptoms, or equivocal duplex findings.

## Discussion

PAAs are frequently bilateral, occurring in up to 50% of patients, and are associated with concomitant aneurysms, particularly abdominal aortic aneurysms, in a significant proportion of cases [2,3]. Although many PAAs are asymptomatic at diagnosis, their natural history is characterized by a high risk of thrombosis and distal embolization, which may result in acute limb ischemia and limb loss if left untreated [10]. Rupture is relatively uncommon in PAAs compared with other aneurysms, but overall morbidity remains substantial due to ischemic complications [2,10].

True SFAAs are uncommon but well-recognized vascular lesions. Contemporary case series have shown that they are encountered infrequently in routine vascular practice and often present late because of their deep anatomical location, allowing progressive enlargement before diagnosis [6,7]. They may present with nonspecific symptoms or complications such as thrombosis, distal embolization, or rupture [4-8]. Compared with PAAs, SFAAs are more likely to present with rupture rather than ischemic complications, particularly when they reach a large size [4,5]. Their rarity and variable presentation make diagnosis challenging and highlight the importance of imaging in evaluation.

The diagnosis of peripheral arterial aneurysms, including PAAs and SFAAs, relies on clinical suspicion supported by imaging. Duplex ultrasonography is typically the first-line modality due to its noninvasive nature and ability to assess aneurysm size, mural thrombus, and distal flow [2,3]. CTA is considered the gold standard for anatomical assessment and operative planning, allowing accurate evaluation of aneurysm extent and distal runoff [3]. Magnetic resonance angiography may be used in selected patients [5].

Management depends on aneurysm size, symptoms, and associated complications. Current guidelines recommend intervention for symptomatic aneurysms and for asymptomatic PAAs greater than 2 cm due to the risk of thromboembolism [3]. SFAAs are generally treated once diagnosed because of their higher risk of rupture [4,5,8]. Both open surgical and endovascular approaches are used depending on patient and anatomical considerations [3,8].

Recent evidence supports individualized treatment selection for popliteal and SFAAs based on symptoms, anatomy, operative risk, distal runoff, and conduit availability. The Society for Vascular Surgery guidelines on PAAs provide evidence-based recommendations regarding screening, indications for intervention, choice of repair strategy, and follow-up [11]. Contemporary studies continue to show that endovascular repair is a feasible minimally invasive option, particularly in selected high-risk patients, but it may be associated with higher early graft thrombosis and reintervention rates compared with open repair [12,13].

Open repair with autologous vein bypass remains a durable option, especially in surgically fit patients with suitable venous conduit, because of its favorable long-term patency and limb-salvage outcomes [11-13]. Open surgical repair remains the gold standard, particularly in fit patients. The preferred technique involves aneurysm exclusion and bypass using the GSV [2,7]. Vein grafts demonstrate favorable long-term outcomes, with reported primary patency rates of approximately 70-80% at five years and 60-70% at 10 years, and secondary patency rates exceeding 80-90% at five years [10,12]. These outcomes are attributed to better resistance to infection and improved hemodynamic compatibility compared with prosthetic grafts [2,10]. Prosthetic grafts, such as polytetrafluoroethylene, are used when suitable vein conduits are unavailable but are associated with inferior durability. Reported primary patency rates are approximately 50-60% at five years, particularly in infrainguinal bypasses [5,14]. Prosthetic grafts also carry higher risks of infection and thrombosis, which may negatively impact limb salvage [5].

Endovascular repair using stent grafts offers a minimally invasive alternative, particularly in high-risk patients. Reported primary patency rates are approximately 75-85% at one year and 60-70% at three years [3,15]. However, long-term durability remains a concern due to complications such as stent fracture, endoleak, and occlusion, especially in the popliteal artery [3,15]. Comparative studies suggest that although early outcomes are favorable, open repair with autologous vein grafts continues to demonstrate superior long-term patency and limb salvage [10,14]. Recent reports on SFAAs also emphasize their rarity, delayed diagnosis, and variable presentation, ranging from limb ischemia to rupture, supporting early imaging and individualized operative planning [16,17].

Although endovascular repair has gained popularity because of its minimally invasive nature and shorter recovery time, its long-term durability remains less established, particularly in younger or surgically fit patients. In contrast, open surgical repair with autologous GSV bypass continues to demonstrate superior long-term primary patency, lower reintervention rates, and more durable limb salvage, especially for complex or large aneurysms with suitable venous conduit. Consequently, current guidelines continue to favor open repair in patients who are acceptable surgical candidates, while reserving endovascular treatment for selected high-risk patients or those with unfavorable operative profiles.

Although aneurysms involving the superficial femoral and popliteal arteries are recognized entities in vascular surgery, giant lesions remain uncommon and may present diagnostic and therapeutic challenges. The educational value of this case lies in the combination of a large aneurysm presenting with chronic ischemic symptoms despite preserved distal runoff, comprehensive preoperative imaging, and successful open reconstruction using a non-reversed in situ autologous GSV bypass. This case reinforces important principles in diagnosis, operative planning, and surgical management rather than presenting a previously unreported pathology.

This case highlights several important clinical lessons. Giant peripheral arterial aneurysms may present with subtle ischemic symptoms despite carrying a substantial risk of thromboembolic complications. Careful vascular examination and prompt CTA are essential for accurate diagnosis and operative planning. In appropriately selected patients, open aneurysm exclusion with autologous GSV bypass remains a durable revascularization strategy with excellent limb-salvage potential.

The main strengths of this report include the description of a rare vascular pathology with detailed imaging, operative technique, and favorable six-month clinical and radiological outcomes. Its principal limitation is the single-patient design, which limits the generalizability of the findings.

## Conclusions

This case illustrates that giant superficial femoral-PAAs may present with chronic ischemic symptoms despite preserved distal runoff. Comprehensive vascular assessment and CTA enabled accurate diagnosis and operative planning. Successful open reconstruction with an autologous GSV bypass resulted in an excellent clinical outcome at six-month follow-up. These findings support current evidence favoring individualized management based on patient and anatomical characteristics.
